# Supplementary material for: Differential Anti-Tumor Effects of IFN-Inducible Chemokines CXCL9, CXCL10, and CXCL11 on a Mouse Squamous Cell Carcinoma Cell Line
Source: Med Sci (Basel). 2023 Apr 25;11(2):31. doi: 10.3390/medsci11020031 (PMC10204432; doi:10.3390/medsci11020031)
Supplement: Supplementary file 1 [file medsci-11-00031-s001.zip › Supplementary_Figure_S1.pdf]

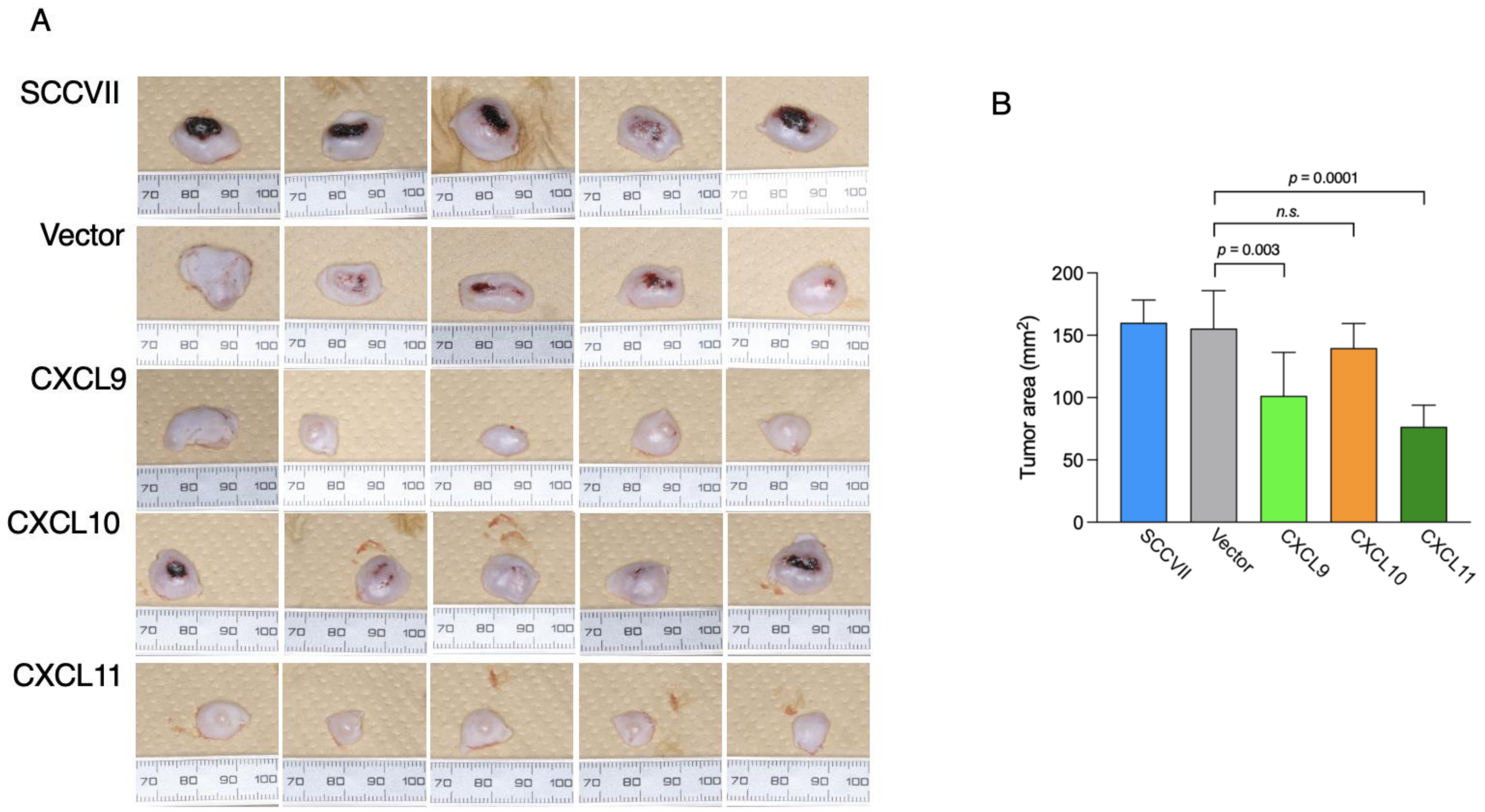


**Figure S1.** Gross observation of the extracted tumor and tumor size.

A. Chemokine-expressing cell lines were subcutaneously transplanted into the back of nude mice, and tumors were extracted three weeks later. Ulcerations caused by overgrowth of tumors were observed in tumors transplanted with parental SCCVII cells, Vector, and CXCL10-expressing cell lines.

B. The size of the extracted tumor was measured in the images, and the area was determined using ImageJ (Version 1.51, National Institutes of Health, Bethesda, MD, USA). Each column and bar represent the mean ± SD (n = 5). Statistically differences in the tumor size relative to the control vector are indicated (one-way ANOVA). "n.s." indicated no statistical significance.
