# Supplementary material for: Differential Anti-Tumor Effects of IFN-Inducible Chemokines CXCL9, CXCL10, and CXCL11 on a Mouse Squamous Cell Carcinoma Cell Line
Source: Med Sci (Basel). 2023 Apr 25;11(2):31. doi: 10.3390/medsci11020031 (PMC10204432; doi:10.3390/medsci11020031)
Supplement: Supplementary file 1 [file medsci-11-00031-s001.zip › Supplementary_Table_S1.pdf]

Table S1. Nucleotide sequences of primers for the construction of chemokine expression vector by the Gateway cloning system

Primer Name	Bases	Nucleotide sequences
attB1 Cxcl9	69	5’-GGGGACAAGTTTGTACAAAAAAGCAGGCTTCGAAGGAGATAGAACC**ATG**AAGTCCGCTGTTCTTTTCCT
attB2 Cxcl9	53	5’-GGGGACCACTTTGTACAAGAAAGCTGGGTC**CTA**TGTAGTCTTCCTTGAACGAC
attB1 Cxcl10	69	5’-GGGGACAAGTTTGTACAAAAAAGCAGGCTTCGAAGGAGATAGAACC**ATG**AACCCAAGTGCTGCCGTCAT
attB2 Cxcl10	53	5’-GGGGACCACTTTGTACAAGAAAGCTGGGTC**CTA**AGGAGCCCTTTTAGACCTTT
attB1 Cxcl11	69	5’-GGGGACAAGTTTGTACAAAAAAGCAGGCTTCGAAGGAGATAGAACC**ATG**AACAGGAAGGTCACAGCCAT
attB2 Cxcl11	53	5’-GGGGACCACTTTGTACAAGAAAGCTGGGTC**CTA**CATGTTTTGACGCCTTAAAA

Red characters indicate the initiation and stop codon, respectively. Blue characters indicate the cording region of the IFN-inducible chemokines.
